# A prognostic model using the neutrophil-albumin ratio and PG-SGA to predict overall survival in advanced palliative lung cancer

**DOI:** 10.1186/s12904-022-00972-x

**Published:** 2022-05-18

**Authors:** Changyan Feng, Huiqing Yu, Haike Lei, Haoyang Cao, Mengting Chen, Shihong Liu

**Affiliations:** 1 Chongqing Key Laboratory of Translational Research for Cancer Metastasis and Individualized Treatment, Chongqing University Cancer Hospital, No. 181 Hanyu Road, Shapingba District, Chongqing, 400030 People’s Republic of China; 2 Chongqing Cancer Multi-omics Big Data Application Engineering Research Center, Chongqing University Cancer Hospital, No. 181 Hanyu Road, Shapingba District, Chongqing, 400030 People’s Republic of China

**Keywords:** Lung cancer, Prognosis, Patient-generated subjective global assessment, Neutrophil-albumin ratio, Palliative

## Abstract

**Objective:**

Inflammation and malnutrition are common in patients with advanced lung cancer undergoing palliative care, and their survival time is limited. In this study, we created a prognostic model using the Inflam-Nutri score to predict the survival of these patients.

**Methods:**

A retrospective cohort study was conducted on 223 patients with advanced, histologically confirmed unresectable lung cancer treated between January 2017 and December 2018. The cutoff values of the neutrophil-albumin ratio (NAR) and Patient-Generated Subjective Global Assessment (PG-SGA) score were determined by the X-tile program. Least absolute shrinkage and selection operator (LASSO) Cox regression and multivariate Cox regression analysis were performed to identify prognostic factors of overall survival (OS). We then established a nomogram model. The model was assessed by a validation cohort of 72 patients treated between January 2019 and December 2019. The predictive accuracy and discriminative ability were assessed by the concordance index (C-index), a plot of the calibration curve and risk group stratification. The clinical usefulness of the nomogram was measured by decision curve analysis (DCA).

**Results:**

The nomogram incorporated stage, supportive care treatment, the NAR and the PG-SGA score. The calibration curve presented good performance in the validation cohorts. The model showed discriminability with a C-index of 0.76 in the training cohort and 0.77 in the validation cohort. DCA demonstrated that the nomogram provided a higher net benefit across a wide, reasonable range of threshold probabilities for predicting OS. The survival curves of different risk groups were clearly separated.

**Conclusions:**

The NAR and PG-SGA scores were independently related to survival. Our prognostic model based on the Inflam-Nutri score could provide prognostic information for advanced palliative lung cancer patients and physicians.

## Introduction

Globally, lung cancer has been the most commonly diagnosed cancer for the last several decades. In 2018, an estimated 2.1 million new lung cancer cases were diagnosed worldwide, and lung cancer is currently the leading cause of cancer death, accounting for nearly 20% [1]. Lung cancer is a major public health issue and places an enormous burden on society in China. It is projected that lung cancer mortality in China will increase by approximately 40% between 2015 and 2030 [2]. The majority of patients present with late-stage disease at the time of initial diagnosis [3]. Despite therapeutic progress, the long-term prognosis remains poor, and the 5-year survival rate is only 19% [4].

Once patients have received an accurate diagnosis of cancer, the next question is often about the likely prognosis. The number of lung cancer treatment strategies has grown in recent years and with that comes new hope for living with advanced lung cancer. For instance, the treatment options for advanced and metastatic non-small-cell lung cancer have dramatically changed; immune checkpoint inhibitors and molecular-targeted therapy have improved patient survival [5, 6]. In palliative care practice, the focus is on predicting how long patients are expected to live rather than predicting their response to further treatment [7]. Deaths occurring within 30 days of chemotherapy are usually recognised as an indicator of the quality of cancer care [8]. At the interface of palliative care, prognostic questions are most relevant to decisions regarding whether to proceed with palliative chemotherapy. When used appropriately, palliative chemotherapy can improve the survival and quality of life of patients with advanced cancer [9]; however, when administered to patients who are near the end of life, even with a relatively good performance status, chemotherapy might adversely affect their quality of life [10].

It is well recognized that prognostic factors are useful in guiding drug selection and monitoring the response to treatment. An adequate evaluation of both the preferences of the patient and prognosis and survival is necessary to determine the optimal treatment strategies, provide assistance for care planning, and efficiently use of available resources [11]. The majority of cancer patients presenting with advanced-stage disease have systemic inflammation [12] and are malnourished, and they have weak immunity due to tumour progression, poor nutritional status, and the side effects of therapy.

Some non-invasive markers, such as the neutrophil–lymphocyte ratio (NLR) [13], neutrophil-albumin ratio (NAR) [14], and the Patient-Generated Subjective Global Assessment (PG-SGA) score [15], can be used to assess the prognosis of many malignant tumours. Although the prognostic influences of the inflammatory response and nutritional status are well established, no risk model based on these scores has been provided, and there remains no widely used prognostic tool for patients with advanced lung cancer undergoing palliative care. Therefore, the aim of this study was to construct a prognostic model based on inflammatory markers and the PG-SGA score in patients with locally advanced or metastatic lung cancer treated with palliative care.

## Methods

### Study design and population

The palliative care unit in Chongqing University Cancer Hospital (CUCH), Chongqing, China, was established to integrate palliative care, including palliative therapy, symptom management, cancer pain management, nutritional therapy, and psychosocial and social support for advanced cancer patients. All patients included in this study were first admitted to the palliative care unit of CUCH between January 2017 and December 2019 with stage III (locally advanced) or IV (metastatic), histologically confirmed lung cancer. Patients referred between January 2017 and December 2018 were included as the main training cohort, and those referred between January 2019 and December 2019 were kept as a separate testing cohort for validation. The exclusion criteria were incomplete relevant laboratory or nutritional data, multiple primary tumours, and previous radical surgery.

Data regarding age, sex, Karnofsky Performance Status (KPS), concomitant disease, histologic classification, tumour stage, treatment after referral to the palliative care unit and albumin level were extracted from medical records. Peripheral blood cell tests, including absolute neutrophil count and lymphocyte count, were administered within 3 days of admission to the palliative care unit. For each patient, clinical and demographic information were extracted from the electronic records of CUCH, and nutrition assessment data were extracted from the database of the Investigation on Nutrition Status and Clinical Outcome of Common Cancers (INSCOC) project in China. The detailed study design of the INSCOC project has been described previously [16, 17]. All patients were recruited according to these data at the time of initiating palliative care.

The serum prognostic markers under investigation were the NLR and NAR. The NLR was defined as the neutrophil count divided by the lymphocyte count [18]. The NAR was defined as neutrophil count divided by albumin level [19]. Nutritional status was evaluated using the score of the PG-SGA [20] by trained nutritionists within 24 h of admission, assessed with a questionnaire about patient weight, food intake, symptoms, functional ability, and metabolic abnormalities along with a detailed physical examination by the physicians [21].Ethics approval was obtained from the local institutional review board. Informed consent was waived due to the retrospective nature of the study. All treatments were performed in accordance with the relevant guidelines and regulations. Overall survival (OS) time was defined as the period from the date of initial treatment in the palliative care unit of CUCH to death or the last follow-up, the last of which was in October 2021.

### Statistical analysis

Data analyses were performed using Statistical Package for the Social Sciences software (SPSS, version 26.0). Descriptive statistics were used to present the characteristics of the patients.

Categorical variables are described as totals and frequencies. The optimal cutoff values for the NLR, NAR and PG-SGA score were calculated by X-tile software (Yale University, New Haven, CT, USA) [22]. Continuous variables such as the NLR, NAR and PG-SGA score were converted to categorical variables using identified cut points.

To avoid over-fitting and the omission of highly related factors, least absolute shrinkage and selection operator (LASSO) Cox regression was carried out using the primary cohort based on the glmnet package [23] in R version 4.1.2 statistical analysis software (http://www.r-project.org). Univariate and multivariate Cox regression analyses were used to investigate hazard ratios (HRs) for the individual putative serum and nutritional markers, and then a prognostic nomogram model was formulated using the rms package [24]. The performance of the nomogram was estimated by the concordance index (C-index). The nomogram was then validated with the validation cohort, and its performance was assessed by a calibration plot and the C-index. Bootstrapping with 1000 resamples was applied to these estimations. During nomogram validation, the total score for each patient in the validation cohort was calculated according to the established nomogram, Cox regression was performed for the cohort using the total score as a factor, and the C-index and calibration curve were derived based on regression analysis.

Clinical usefulness and net benefit were estimated with decision curve analysis (DCA) [25]. Different combinations of stage, NAR and PG-SGA score were also evaluated by DCA together with the nomogram by comparing observed 1-year event rates with predictions from the final model.

To demonstrate the predictive performance of the combined model, patients were subdivided equally into three subgroups according to the total score calculated by the nomogram for the training cohort, which were also used to divide the validation cohort. Subsequently, the survival curves for different risk groups were generated using the Kaplan–Meier method, and the differences in survival between groups were compared using the log-rank test. All of the tests were 2-sided, and *P* < 0.05 was considered statistically significant.

**Fig. 1:**
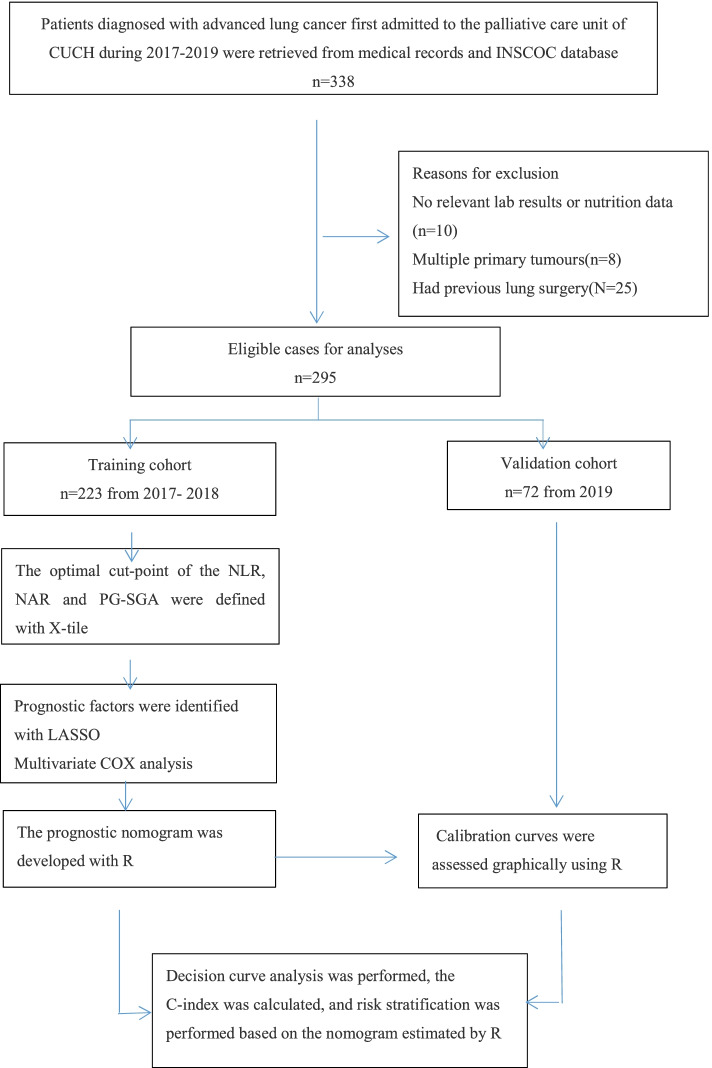
Flow diagram of patient recruitment, data collection and analyses

The flow diagram of patient recruitment, data collection and analyses is shown in Fig. 1.

## Results

### Baseline clinical characteristics

In total, 338 patients with histologically confirmed, advanced lung cancer were referred to the palliative care unit of CUCH between January 2017 and December 2019. Of these, to avoid the potential bias of tumour load, 25 patients who had previously undergone radical surgery were excluded, 8 patients were excluded due to the simultaneous presence of multiple primary malignancies, and 10 patients with incomplete relevant laboratory or nutritional data were excluded.

Finally, 295 patients were eligible for analysis. Sufficient data were available to include 223 patients (164 males and 59 females) in the main training cohort, with a median follow-up of 34.9 months, in which time 189 patients died. For the validation cohort, we included 72 eligible patients (56 males and 16 females). The median follow-up time was 30.4 months, and 63 patients died during follow-up. The characteristics of the patients in the main training and validation cohorts are summarized in Table 1. The median survival time for all patients was 7.1 (range, 5.5–8.7) months. A total of 39 (13.2%) and 256 (86.8%) patients had clinical stages III and IV cancer, respectively. Palliative care treatments included palliative chemoradiotherapy in 42.4% of the patients and targeted therapy or immunotherapy in 25.7%, while 31.9% only received supportive care due to a poor performance status or patient preference.

**Table 1:** Demographics and clinical characteristics of patients with palliatively treated lung cancer

Demographic or Characteristic	Main Training Cohort (*n* = 223)		Validation Cohort (*n* = 72)	
	No. of Patients	%	No. of Patients	%
Sex				
Male	164	73.5	56	77.7
Female	59	26.5	16	22.3
Age, y				
< 60	89	39.9	28	38.9
≥ 60	134	60.1	44	61.1
KPS, %				
< 70	63	28.3	19	26.4
≥ 70	160	71.7	53	73.6
Tumour histology				
Squamous carcinoma	67	30.1	16	22.2
Adenocarcinoma	122	54.7	44	61.1
Small cell lung cancer	34	15.2	12	16.7
Clinical stage				
Stage III	28	12.6	11	15.3
Stage IV	195	87.4	61	84.1
Therapy method				
Palliative chemoradiotherapy	92	41.3	33	45.9
Targeted or immunotherapy	56	25.1	20	27.7
Supportive care	75	33.6	19	26.4
Loss of appetite				
No	126	56.5	42	58.3
Yes	97	43.5	30	41.7
Cancer pain				
No	195	87.4	58	80.6
Yes	28	12.6	14	19.4
Diarrhoea				
No	211	94.6	69	95.8
Yes	12	5.4	3	4.2
Nausea				
No	183	82.1	61	84.7
Yes	40	17.9	11	15.3
Vomiting				
No	190	85.2	58	80.6
Yes	33	14.8	14	19.4
NLR				
Median	4.5		4.2	
IQR	2.7–7.7		2.3–8.4	
NAR				
Median	0.13		0.13	
IQR	0.08–0.22		0.08–0.21	
PG-SGA score				
Median	12		13	
IQR	8–15		8–16	

*Abbreviations:* *KPS* Karnofsky Performance Status, *NLR* Neutrophil-to-lymphocyte Ratio, *NAR* Neutrophil-albumin Ratio, *PG-SGA* Patient-Generated Subjective Global Assessment, *IQR* Interquartile Range

**Fig. 2:**
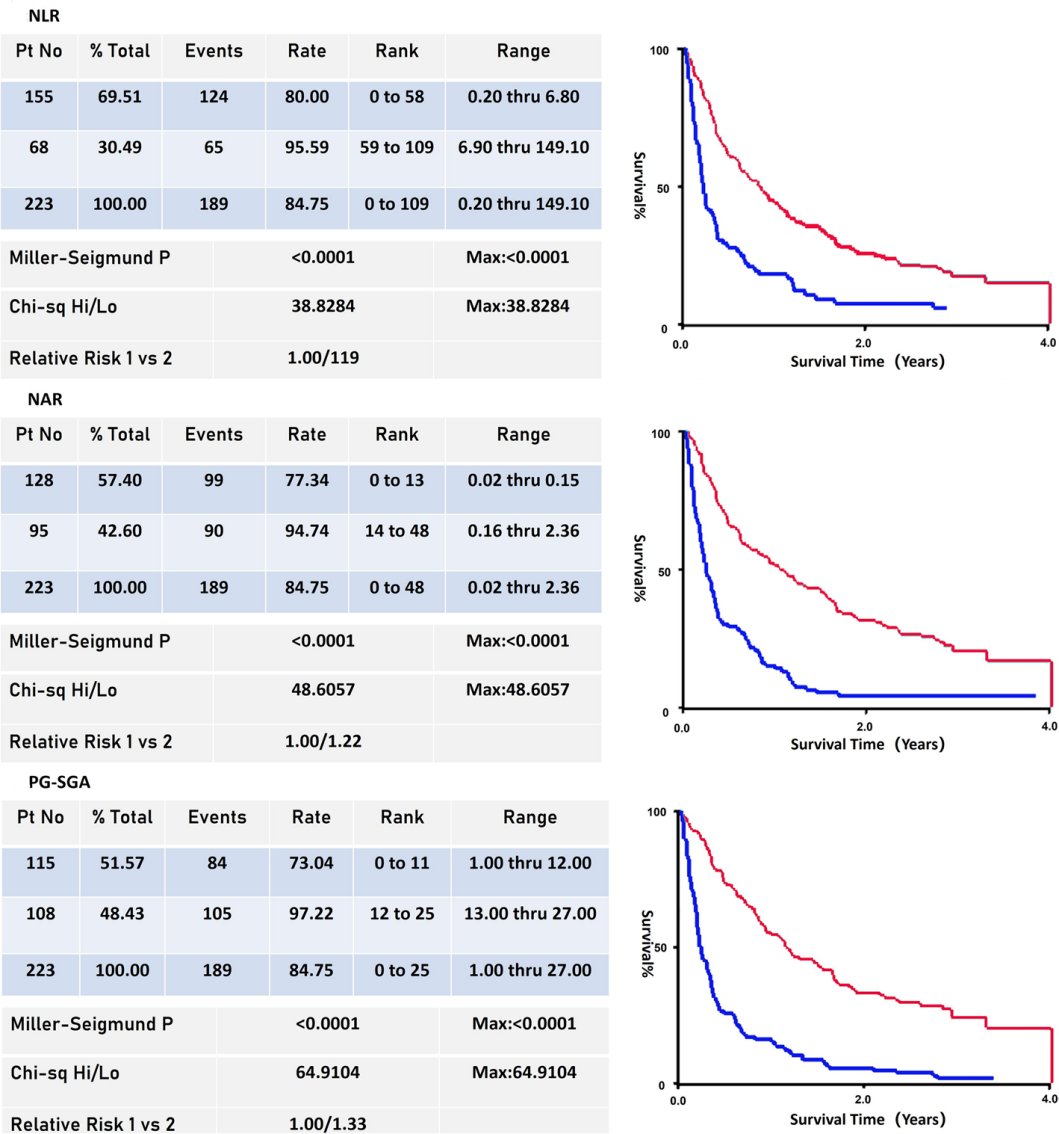
Determination of cutoff values for NLR, NAR and PG-SGA score in the training cohort and associated survival analyses. OS was analysed with X-tile to determine the optimal cutoff values for the NLR, NAR and PG-SGA score: 6.8 (χ^2^ = 38.828, *P* < 0.001), 0.15 (χ^2^ = 48.606, *P* < 0.001), and 12 (χ^2^ = 64.910, *P* < 0.001), respectively

In the main training cohort, the cutoff values of the NLR, NAR and PG-SGA score for eligible patients were determined by the X-tile program to be 6.8, 0.15 and 12, respectively (Fig. 2). The χ^2^ log-rank values of the NLR, NAR and PG-SGA score were 38.828, 48.606 and 64.910, respectively. The patients were divided into pairs of groups for further analysis (NLR ≤ 6.8 and NLR > 6.8; NAR ≤ 0.15 and NAR > 0.15; PG-SGA score ≤ 12 and PG-SGA score > 12).

### Prognostic factor selection

**Fig. 3:**
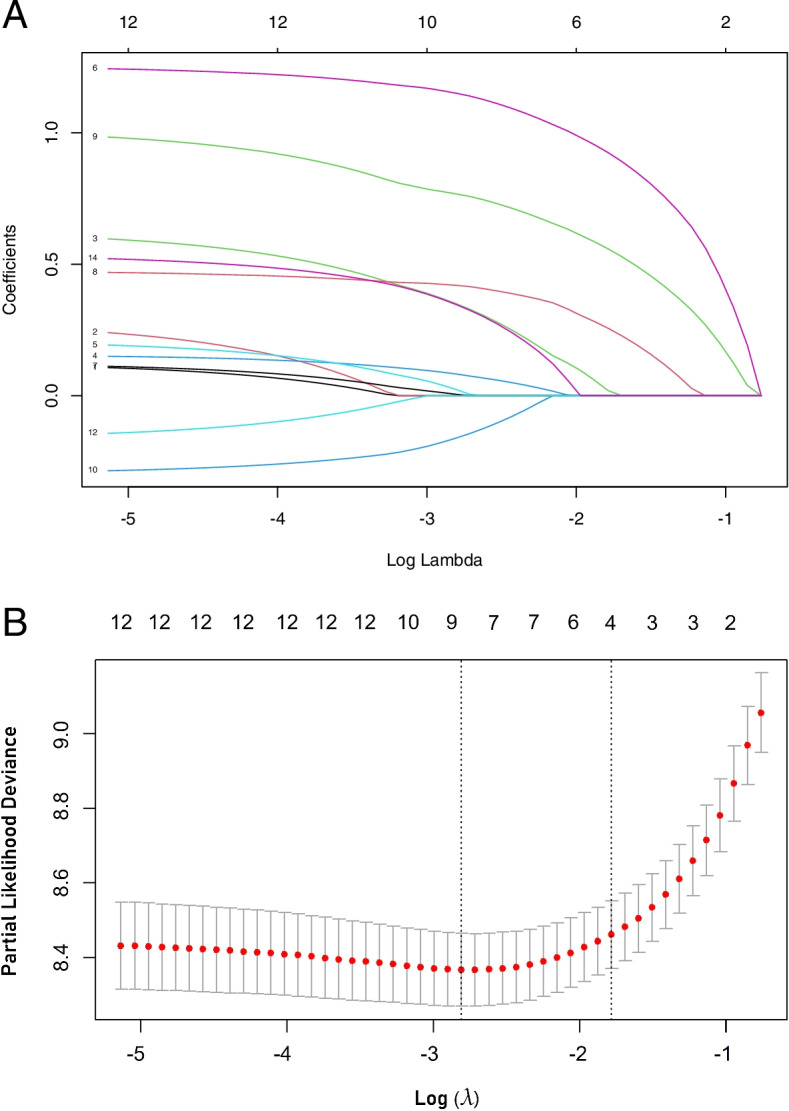
Construction of a predictive model from the 12 prognostic factors. **A**: LASSO coefficient profiles of the 12 prognostic factors. **B**: Four prognostic factors selected using LASSO Cox regression analysis. The two dotted vertical lines were drawn at the optimal scores by minimum criteria and 1-s.e. criteria (the minimum criteria included sex, stage, appetite, cancer pain, supportive care, NLR, NAR, PG-SGA and histology; the 1-s.e. criteria included stage, supportive care treatment, NAR and PG-SGA)

LASSO analysis was performed to identify robust markers. All available clinical indicators, including clinicopathological features and nutritional markers (Table 1), were subjected to LASSO Cox regression, and a significant correlation was observed between sex, stage, appetite, cancer pain, supportive care treatment, NLR, NAR, PG-SGA and histology (Fig. 3A). Further disciplinary regression was performed, and the 1-s.e. criteria, stage, supportive care treatment, NAR and PG-SGA were identified as independent factors for prognosis in patients with advanced palliative lung cancer (Fig. 3B).

Both univariate and multivariate analyses including all clinical variables mentioned above were conducted with a Cox proportional hazards regression model. Univariate analysis of the data from the training cohort revealed the significant association of 9 variables, including age (*P* = 0.035), KPS (*P* < 0.001), stage (*P* = 0.013), loss of appetite (*P* < 0.001), cancer pain (*P* = 0.002), supportive care treatment (*P* < 0.001), NLR (*P* < 0.001), NAR (*P* < 0.001) and PG-SGA score (*P* < 0.001), with OS. Furthermore, multivariate Cox regression using these 9 variables was performed to identify any markers that were independent predictors of OS. The results of this analysis can be found in Table 2. The results showed that stage IV (*P* = 0.014), supportive care treatment (*P* < 0.001), NAR > 0.15 (*P* = 0.014) and PG-SGA score > 12 (*P* < 0.001) remained independent predictors of OS.

**Table 2:** Univariate and multivariate Cox regression analysis of factors associated with OS for the main training cohort

Variable	Univariate	*P* value	Multivariate	*P* value
	HR (95% CI)		HR (95% CI)	
**Significant factors**				
Age ≥ 60 years (vs. < 60 years)	1.383 (1.023–1.871)	0.035	1.215 (0.885–1.669)	0.229
KPS < 70 (vs. ≥ 70)	2.046 (1.501–2.789)	< 0.001	0.750 (0.524–1.074)	0.116
Stage IV (vs. IIIB)	1.784 (1.131–2.814)	0.013	1.794 (1.124–2.863)	0.014
Loss of appetite	2.123 (1.586–2.842)	< 0.001	1.192 (0.861–1.651)	0.290
Cancer pain	1.929 (1.268–2.932)	0.002	1.354 (0.869–2.108)	0.180
Treatment method, supportive care (vs. others)	5.551 (3.939–7.709)	< 0.001	3.457 (2.383–5.016)	< 0.001
NLR, > 6.8 (vs. ≤ 6.8)	2.619 (1.922–3.569)	< 0.001	1.082 (0.713–1.642)	0.711
NAR, > 0.15 (vs. ≤ 0.15)	2.831 (2.102–3.813)	< 0.001	1.674 (1.108–2.527)	0.014
PG-SGA score, > 12 (vs. ≤ 12)	3.348 (2.481–4.519)	< 0.001	2.396 (1.665–3.446)	< 0.001
**Nonsignificant factors**				
Sex, female (vs. male)	1.299 (0.931–1.813)	0.123		
Histology, small-cell lung cancer (vs. other)	1.329 (0.903–1.956)	0.150		
Diarrhoea	1.625 (0.902–2.928)	0.106		
Nausea	1.015 (0.700–1.472)	0.938		
Vomiting	1.223 (0.823–1.818)	0.319		

*Abbreviations: CI* Confidence Interval, *HR* Hazard Ratio, *KPS* Karnofsky Performance Status, *NLR* Neutrophil-to-lymphocyte Ratio, *NAR* Neutrophil-albumin Ratio, *PG-SGA* Patient-Generated Subjective Global Assessment

### Prognostic nomogram model for OS

**Fig. 4:**
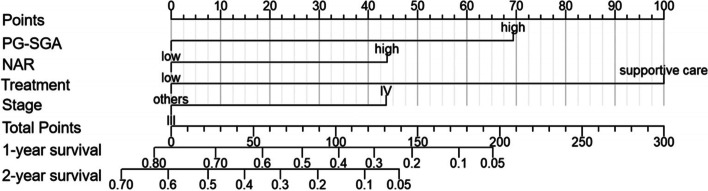
Nomogram model for predicting 1-year and 2-year overall survival (OS) in palliative care lung cancer patients in the main training cohort

As stage, supportive care treatment, NAR and PG-SGA score were found to be independent predictors of survival, a nomogram for predicting the survival of palliative care patients with advanced lung cancer was built based on the values of these four independent prognostic markers from the training cohort (Fig. 4). The C-index of the nomogram for predicting OS was 0.76 (95% CI, 0.74 to 0.78). Then, these markers were used to form a prognostic scoring system using the following maximum scores: stage IV = 44, supportive care treatment = 100, NAR (> 0.15) = 44, and PG-SGA score (> 12) = 69. The sum of the scores corresponding to these factors was used to estimate a particular patient’s 1- and 2-year survival probability. The total prognostic score ranges from 0 to 257, and a higher score implies a poorer prognosis.

### Validation of the nomogram

**Fig. 5:**
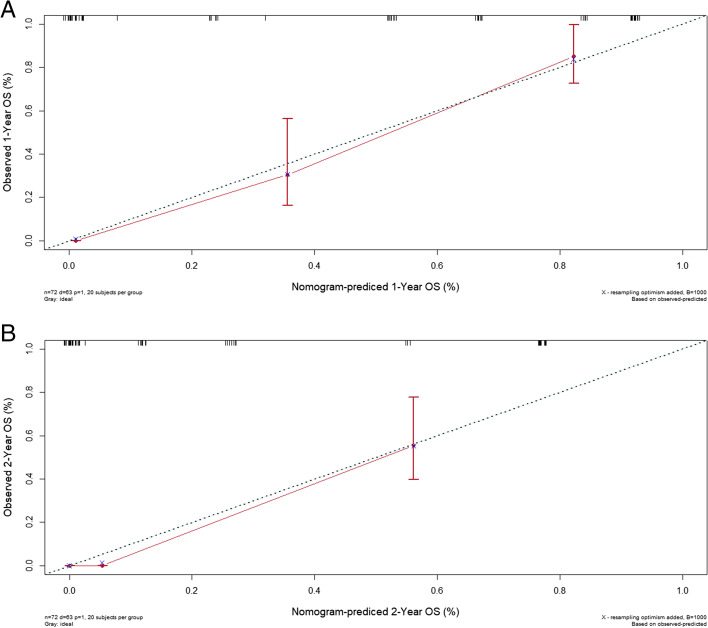
Calibration curves used to compare the nomogram-predicted and actual measured survival probabilities at 1 year and 2 years for the validation cohort (5**A** and 5**B**, respectively). The y-axis represents the actual measured survival probability, and the x-axis represents the nomogram-predicted survival probability. The diagonal dotted line represents a perfect prediction by an ideal model. The red solid line represents the performance of the nomogram; a closer fit to the diagonal dotted line represents a better prediction

In the validation cohort, the median OS time was 8.5 months (range, 4.3 to 12.6 months). The C-index for predicted OS was 0.77 (95% CI, 0.71 to 0.83). The calibration curve indicated good agreement for 1-year and 2-year OS between the nomogram-predicted and observed probability of survival for palliative care lung cancer patients (Fig. 5).

**Fig. 6:**
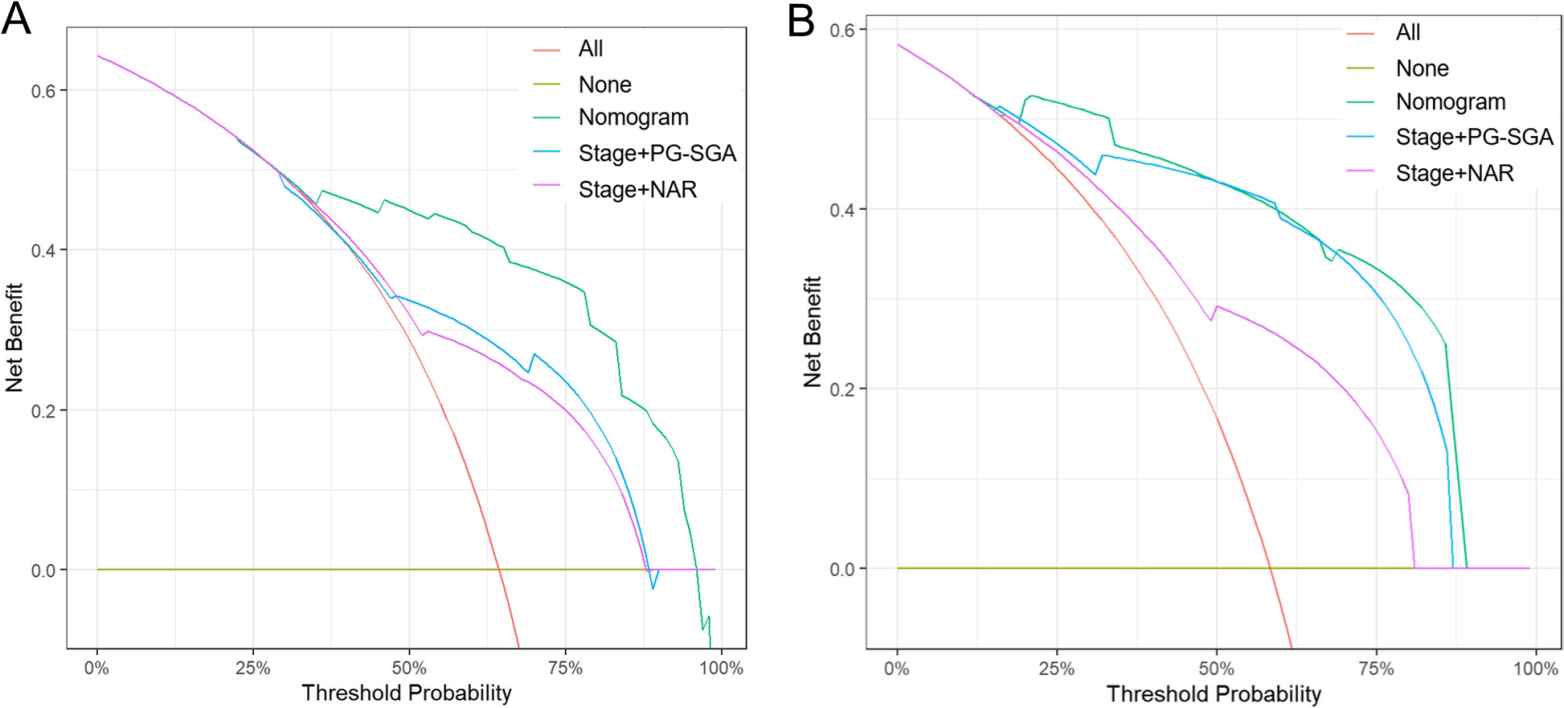
Decision curve analysis for the prediction nomogram model for 1-year OS. **A**: Decision curves for the main training cohort; **B**: Decision curves for the validation cohort

DCA was used to facilitate the comparison between different prediction models and show the clinical usefulness of each. The x-axis shows the range of threshold probabilities, and the y-axis measures the net benefit [26]. A DCA plot for the prediction model for 1-year OS with the main training cohort and validation cohort is presented in Fig. 6A and B, respectively. In this analysis, the figure illustrates that the NAR-PG-SGA nomogram risk score provides a larger net benefit across the range of survival risks than both the stage + PG-SGA and stage + NAR risk scores.

**Fig. 7:**
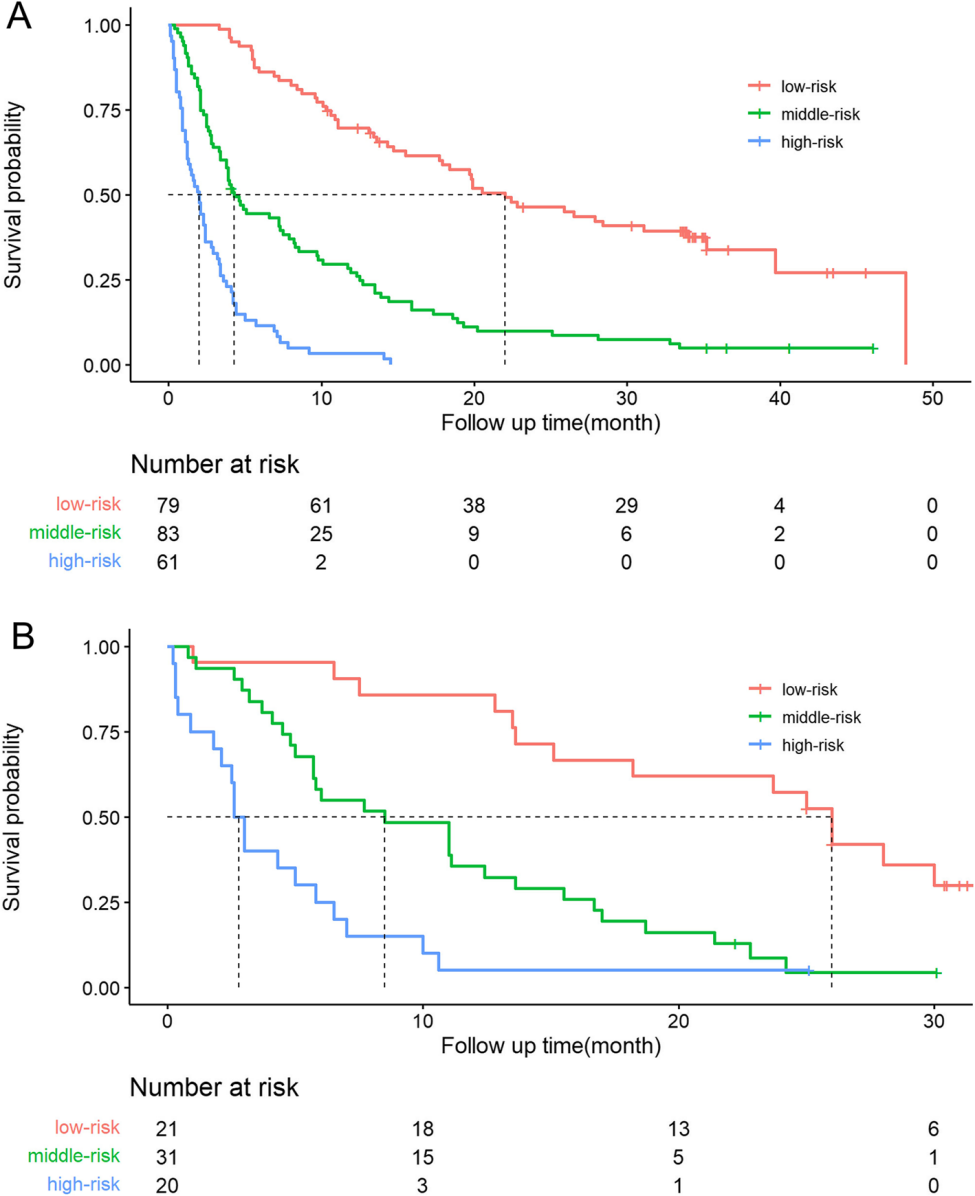
Kaplan–Meier survival curves for the training cohort (**A**) and validation cohort (**B**) showing the OS probability stratified by different levels of risk

Risk stratification based on the nomogram was conducted to show the clinical usefulness of the model. Patients in the training cohort were sorted by the total score calculated by the nomogram and then divided into three equal subgroups based on the risk profile: low-risk (≤ 44 points), middle-risk (> 45 and ≤ 157 points) and high-risk (> 157 points); each subgroup represents a distinct prognosis. Similarly, the validation cohort was divided into three groups using the same cutoff values as in the training cohort. The median OS times in the three risk groups in the training cohort were 22, 4.3 and 2 months (*P* < 0.001), and those in the validation cohort were 26, 8.5 and 2.8 months (*P* < 0.001). The Kaplan–Meier survival curves for both the training and validation cohorts were clearly separated (Fig. 7A and B).

## Discussion

To our knowledge, this is the first study to demonstrate that the NAR has significant prognostic value for patients with advanced lung cancer treated with palliative care. In addition, we confirmed the finding of a previous study, which showed that the PG-SGA score also has prognostic value for these patients. The findings indicate that a higher NLR, NAR, or PG-SGA value indicates a worse outcome. Multivariate Cox regression analysis allowed us to identify stage, supportive care treatment, NAR and PG-SGA score as independent predictors of survival, making them eligible for inclusion in our Inflam-Nutri model. We demonstrated that this novel scoring system can stratify advanced lung cancer patients in the palliative care setting into distinct prognostic groups, allowing for an improved estimate of survival. Furthermore, a separate cohort of patients was used to successfully validate the prognostic value of the Inflam-Nutri model.

Cancer-related inflammation plays an important role in tumour progression by fostering cancer cell proliferation, invasion, and metastasis and affecting the tumour response to systemic therapies [27], resulting in poor survival [28]. Tan [29] et al. confirmed the positive relationship between nutritional status, inflammatory markers, and survival in patients with advanced cancer. There is also good evidence that C-reactive protein (CRP) and the NLR are both sensitive and reliable markers of systemic inflammation in cancer patients [30–32]; unfortunately, CRP measurements are not routinely performed for our palliative cancer patients.

The NLR and NAR are inexpensive and easily accessible serum markers of systemic inflammation. Diem S et al.found that an elevated NLR was associated with worse OS and lower response rates in patients with non-small-cell lung cancer (NSCLC) treated with nivolumab [33]. However, in the multivariate analysis, we did not observe that the NLR contributed significantly to the prediction of OS, possibly because only a small number of patients received immunotherapy in our study. Furthermore, our findings are in line with previous observations showing that the pretreatment NAR significantly predicted the outcome of pathological complete response to neoadjuvant chemotherapy in rectal cancer [34] and that it can predict overall survival for pancreatic cancer patients receiving palliative care [35].

Because of the negative impact on clinical outcome in advanced cancer, nutritional assessment serves as the basis for a malnutrition diagnosis and includes determination of the cause, severity and type of malnutrition [36]. The PG-SGA score is an internationally recognized method for proactive risk screening, assessment, monitoring and triaging for interventions in patients with cancer [20]. It is administered via a questionnaire that is answered by both the patient and doctors and includes questions on weight changes, intake conditions, symptoms affecting eating, functional ability, and metabolic abnormalities, along with a detailed physical examination by a physician. The PG-SGA questions can be quickly answered by the patient, generally in less than 5 min. It is easy to use and does not require any other equipment; however, the PG-SGA is subjective and can be affected by evaluator bias [37]. Training may address potential barriers [38], and in our study, the PG-SGA was administered by dietitians who were well trained and had experience with this clinical instrument. Furthermore, the PG-SGA can identify nutrition impact symptoms, which usually occur during the cancer continuum. Interestingly, of all the symptoms examined by the PG-SGA in our study, none was associated with increased survival, whereas another study showed that xerostomia was associated with reduced survival [15]. This may be because our study focused on the early delivery of patient care by specialist palliative care teams alongside tumour treatment to promote patient-centred care, including basic symptom management, inflammation and nutritional status assessment. In addition, completing the PG-SGA form may increase the patient’s awareness of his or her malnutrition risk [20] and facilitate proactive, early malnutrition prevention.

In this study, the cutoff point that best predicted the risk of death was a PG-SGA score > 12 points. However, in Wiegert EVM’s [15] study, the cutoff point that best predicted the risk of death was a PG-SGA score ≥ 20 points, which is much higher than that in our study. Furthermore, a PG-SGA score ≥ 9 points is frequently used as a cutoff to indicate a critical need for improved symptom management or nutrition intervention [20]. Our results may suggest that defining the cutoff point as ≥ 9 points might not be suitable in clinical practice to triage advanced lung cancer patients treated with palliative care, and further work should be considered to clarify the appropriate thresholds.

The NAR and PG-SGA scores have significant prognostic value for advanced lung cancer patients treated with integrated palliative care. Multivariate Cox regression analysis allowed us to identify the NAR and PG-SGA score as independent predictors of survival, making them eligible for inclusion in our Inflam-Nutri prediction model. Our results further indicate that the median OS and survival rates were all significantly different. However, research on the survival prognostic value of inflammatory- and nutrition-based indicators in advanced lung cancer patients receiving palliative care is limited. Additional external validation is warranted to further confirm our conclusion. Moreover, defining the ability of the NAR-PG-SGA model to predict the response to palliative antitumour therapy or to monitor the response to cancer treatments would be of great interest.

There were some potential limitations in this study. First, the present study was a retrospective analysis planned by a single institution. Second, the study enrolled a relatively small sample size, which might be insufficient for achieving convincing results. In addition, since we involved patients who underwent optimal support therapy with palliative treatment for tumours, selection bias may have been present. Accordingly, although promising preliminary data have been shown for the Inflam-Nutri score, further multicentre prospective studies with larger sample sizes are required to confirm the present hypothesis.

## Conclusions

This retrospective study developed a nomogram model including the NAR and PG-SGA score along with stage and supportive care treatment as predictive factors for advanced lung cancer patients treated with integrated palliative care. Based on the four variables, a prognostic model was developed that enabled suitable risk classification and might aid clinicians in making decisions regarding palliative or support therapy by offering more accurate survival estimates. Our findings highlight the potential for using the NAR in conjunction with the PG-SGA score as a routine marker of prognosis in patients with advanced cancer in palliative care.

## Data Availability

The datasets generated and/or analysed during the current study are not publicly available due to privacy or ethical restrictions but are available from the corresponding author upon reasonable request.

## Abbreviations

NLR: Neutrophil-to-lymphocyte ratio
NAR: Neutrophil-albumin ratio
PG-SGA: Patient-Generated Subjective Global Assessment
ROC: Receiver operating characteristic
OS: Overall survival
CUCH: Chongqing University Cancer Hospital
KPS: Karnofsky performance status
HR: Hazard ratio
CI: Confidence interval
AUC: Area under the curve
CRP: C-reactive protein
DCA: Decision curve analysis
C-index: Concordance index
IQR: Interquartile range
LASSO: Least absolute shrinkage and selection operator
